# Remimazolam induced cognitive dysfunction in mice via glutamate excitotoxicity

**DOI:** 10.1515/tnsci-2022-0220

**Published:** 2022-05-31

**Authors:** Xin-hua Zhou, Cheng-cheng Zhang, Ling Wang, Shan-liang Jin

**Affiliations:** Department of Anesthesiology, Shanghai Ninth People’s Hospital, Shanghai Jiao Tong University School of Medicine, No. 639, Zhizaoju Road, Shanghai 201900, China; Department of Anesthesiology, Changhai Hospital, The Naval Medical University, Shanghai 200433, China

**Keywords:** anesthetic, spatial memory, cognitive functions, memory dysfunction, neuronal apoptosis

## Abstract

**Objective:**

Several lines of evidence demonstrated the role of anesthetic drugs in cognitive functions. Some anesthetic agents have been confirmed to be associated with long-term spatial memory and learning in aged animal models.

**Methods:**

C57BL/6 mice were divided into four different groups based on different concentrations of remimazolam treatments. Behavioral phenotype was observed by open field, rota rod, Morris water maze, and elevated plus maze test. Western blot was performed to see the expression pattern of different proteins. Confocal microscopy images were taken for neuronal and glial cells to see the effect of remimazolam on CNS cells.

**Results:**

We showed that remimazolam, a new anesthetic drug, impaired cognitive behavior. Repetitive doses of remimazolam have been found to induce neuronal loss with a significant change in morphology. Here, we showed that a higher concentration of remimazolam had a significant effect on CNS cell activation. We showed that remimazolam caused memory dysfunction by inducing neuronal apoptosis via glutamate excitotoxicity. It also exhibited amyloid β plaque in the brain via abnormal phosphorylation of tau protein. Remimazolam-mediated regulation of glial cells in mouse cortex was observed and robust activation of astrocytes and microglial cells was found. Finally, we assessed the behavioral phenotype of mice and found that treatment with remimazolam induced significant behavioral changes and memory dysfunction.

**Conclusions:**

This study provides insight into the mechanism of anesthetic drug-induced memory deficits and may help improve the therapeutic effects of anesthesia agents in clinical applications.

## Graphical abstract

**Figure:**
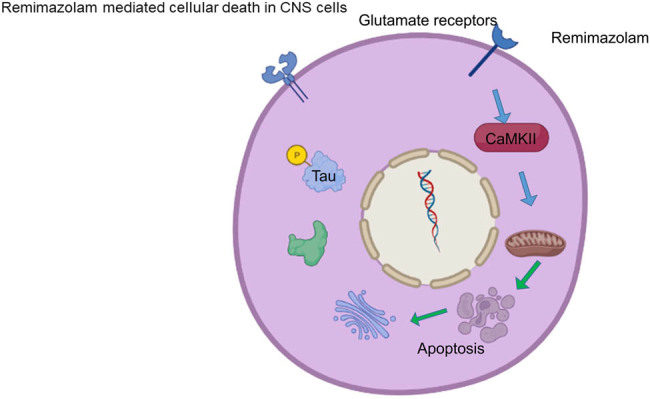

## 1 Introduction

The use of anesthetic medications has made it possible to execute diagnosis and complicated surgeries. Previously, it was thought that general anesthetic drugs produced reversible and temporary effects on central nervous system (CNS), and after discontinuation of drugs the CNS returned to its original form [1]. Currently, it is known that anesthesia produced significant and long-lasting cellular alterations [2]. In young and older adults, anesthetic drugs have been responsible for causing CNS dysfunctions [3]. Astrocyte activation is a common biomarker of neurodegenerative diseases [4,5]. Microglial activation has also been reported in pathological conditions [6]. Most of the anesthetic drugs are *N*-methyl-d-aspartate (NMDA) antagonists and γ-aminobutyric acid (GABA)-A agonists [7]. Previous studies had reported that excessive stimulation of type-A GABA receptors and transient blockade of NMDA receptors triggered the apoptosis of neurons during the brain growth-spurt phase [8–10] and also produced cognitive deficits [11,12]. Spatial learning function was impaired in adulthood when NMDA antagonists were administered during the neonatal phase of rats [13].

Several lines of evidence demonstrated the role of anesthetic drugs that lead to the impairment in memory and cognitive functions [11,14–16]. Researchers demonstrated that ketamine induced neurodegeneration in the developing brain hippocampus and persistent impairments in learning and memory, linked with PKC-ERK (protein kinase C-extracellular signal-regulated kinase) signaling pathway in rats [11]. Clinical reports also showed cognitive impairment following general anesthesia and surgery in middle-aged and older patients, which persisted for weeks to months, respectively [17]. Clinical and preclinical studies showed that anesthetic agents stimulate/initiate neuropathogenesis of Alzheimer’s disease (AD) by inducing hyper-phosphorylation of tau protein followed by deposition of β‑amyloid proteins [18–20]. Glutamate excitotoxicity is one of the common factors of neuronal death due to the buildup of extracellular glutamate concentrations, which resulted in the activation of glutamate receptors (NMDA, AMPA) that further facilitates the influx of excessive ions and leads to cell death [21]. Calcium ions use glutamate receptors and influx inside the cells, and thereby activate calcium-dependent protein kinase (CaMKII). It refers to the pathological condition in which several changes in cellular events take place.

Remimazolam is a novel ester-type benzodiazepine drug having ultra-fast action. It is used intravenously to produce sedation in short medical procedures and induce and maintain general anesthesia [22,23]. It is recently approved for procedural and general anesthesia [24,25].

The present study evaluated the effects of remimazolam on spatial learning and memory. Neurodegeneration and apoptosis related to memory deficits will also be studied. In this study, we found that repetitive doses of remimazolam lead to pathological condition in the brain. We reported that remimazolam produced memory dysfunction by inducing neuronal apoptosis via glutamate excitotoxicity and exhibited amyloid β plaque in brain via abnormal phosphorylation of tau protein. This study also showed the remimazolam mediated regulation of glial cells in mouse cortex, and showed that this drug induced the activation of astrocytes and microglial cells. This study provides insights into the mechanism of anesthetic drug-induced memory deficits and helps improve the therapeutic effects of anesthetic agents.

## 2 Materials and methods

### 2.1 Animals

All animals were maintained in a facility with a light–dark cycle. To avoid gender effects, we only used male mice for behavioral studies and other experiments. All mice were divided into four groups for each experiment, i.e., control group and drug-treated group. Low (10 mg/kg), moderate (15 mg/kg), and high concentrations (20 mg/kg) of remimazolam were used.


**Ethical approval:** The research related to animals’ use has been complied with all the relevant national regulations and institutional policies for the care and use of animals. The experimental procedures were approved by the Animal Care Committee of SPF (Beijing) Biotechnology Co., Ltd. (Beijing, China) (Approval No: AW2021070602).

### 2.2 Hematoxylin and eosin (H&E) staining for neuronal morphology

For histological studies, mouse brain was extracted and fixed in 10% neutral buffered formalin. Brain tissues were embedded in paraffin. Several sections (6 μm) were made from paraffin block and stained with H&E staining. We used mouse cortex region to study the neuronal morphology. All sections of H&E staining were observed using light microscope.

### 2.3 Western blotting

Lysis of cells was performed using lysis buffer (200 μL/well). Constituents of lysis buffer were NaCl (150 mM), Tris (pH 7.6, 50 mM), Triton X-100 (1%), including phosphatase and protease inhibitors. Extracted proteins (40 μg of the total proteins) were separated by performing SDS–PAGE (7.5%). Procedure of western blotting was carried out as described by Waraich et al. and Run et al. [26,27]. Following antibodies were used for western blotting: anti-GFAP, Abcam (ab7260); anti-Oligo2, Abcam (ab136253); anti-Iba1, Abcam (ab5076); anti-beta actin, Abcam (ab115777); anti-caspase-3, Abcam (ab13847); anti-cleave caspase-3, Abcam (ab32042); anti-caspase-9, Abcam, (ab202068); anti-cleave caspase-9, Abcam (ab25758); anti-tau, Abcam (ab76128); anti-tau phospho, Abcam (ab109390); anti-CaMKII, Abcam (ab22609); and anti-CaMKII phospho, Abcam (ab171095). Samples of protein were transferred to nitrocellulose membrane post-electrophoresis. Membrane was blocked by bovine serum albumin or skim milk for 2 h and later incubated overnight with primary antibody (Ab) at 4°C. After washing, membrane was incubated with secondary-Ab (anti-rabbit/mouse Ig-G conjugated to horseradish peroxidase) for 1 h at room temperature. Enhanced chemiluminescence was carried out to visualize protein expression. All western blot experiments were in triplicate. ImageJ was used for protein quantification.

### 2.4 Confocal microscopy

Following steps were performed during preparation of samples: fixation, permeabilization, labeling, and mounting. After extracting the mouse brain, it was fixed in 4% paraformaldehyde at 4°C for 24 h, followed by 30% sucrose solution storage for 24 h at room temperature. Brain tissue was further embedded in cryofreezing medium and stored at −80°C for 1 day. For microscopic study 20 μm thick sections of brain tissues were made. Following antibodies were used for immunofluorescence GFAP antibody, Abcam (ab7260, 1:800); Oligo2 antibody, Abcam (ab136253, 1:1,000); and Iba1 antibody, Abcam (ab5076, 1:750). Before proceeding to primary antibodies, sections were washed three times with phosphate buffer saline buffer for 5 min each. Sections were incubated overnight in primary antibodies at 4°C, followed by appropriate conjugated secondary antibodies for 1 h. Zeiss LSM700 was used to obtain images from the cortex region.

### 2.5 Behavioral experiments

#### 2.5.1 Open field test

Open field test is mostly used to monitor locomotor activity, exploration, and motor depressant effects [28]. It is also used to examine anxiety-like behavior. Typically rodents preferred to move closer to walls (thigmotaxis) but not in central or lit arena of the apparatus. Anxiolytic effects increased the time spent in central area. The open field used in the current study had square arena (76 cm × 76 cm) with walls of 42 cm height. The floor of the apparatus consisted of 25 equal sized squares. During monitoring of activity, the animal was placed in the central box of the apparatus. Activity was counted for 5 min as number of square crossings. Square crossed with all four paws was considered as one. Latency (time taken to move from starting point) was also monitored.

#### 2.5.2 Elevated plus maze (EPM) test

The EPM apparatus and procedure were same as described previously by Ali et al. [29]. This maze apparatus had plus shape and consisted of four arms of 50 cm length and 10 cm width. Two arms were closed with side walls of 15 cm height, and others two arms were open having no side walls. The maze had a height of 60 cm from the ground. During monitoring of anxiety, an animal was placed in the center point of the maze and allowed to explore the entire apparatus for 5 min. Number of entries and time passed in the open arm were monitored during the study.

#### 2.5.3 Rota rod test

Rota rod was used to monitor motor coordination as described earlier by Haleem et al. [30]. The apparatus with drum having diameter of 7 cm was set to 2–20 rpm speed during training phase. During test phase, a fixed speed of 20 rpm was adjusted. To measure the motor coordination, latency to fall was monitored during the test, and cut-off time was 150 s.

#### 2.5.4 Morris water maze test

Morris water maze apparatus and procedure were same as demonstrated previously by Salman et al. [31]. The apparatus was a circular pool (diameter 132 cm, height 60 cm) as introduced by Morris, made of white plastic. Water maze was filled with water (opacified by milk) to 30 cm depth. The pool was divided equally into four quadrants (east, north, west, and south) virtually. A platform (10 cm × 10 cm) was placed at central point of the north quadrant. Cut-off time was 120 s, and if animals were unable to find the platform, they were guided. This test was used to monitor spatial working memory. First animals were trained in water maze, and 2 h post-training learning acquisition was investigated. Memory retention was evaluated 24 h post-learning acquisition. Time taken to locate the hidden platform was noted during the test.

## 3 Statistical analysis

All experiments in this study were performed three times. Data were analyzed by using GraphPad Prism. Multiple group differences were analyzed using one-way ANOVA. *P* value <0.05 was considered to be statistically significant.

## 4 Results

### 4.1 Remimazolam induced neuronal loss in mouse cortex

**Figure 1:**
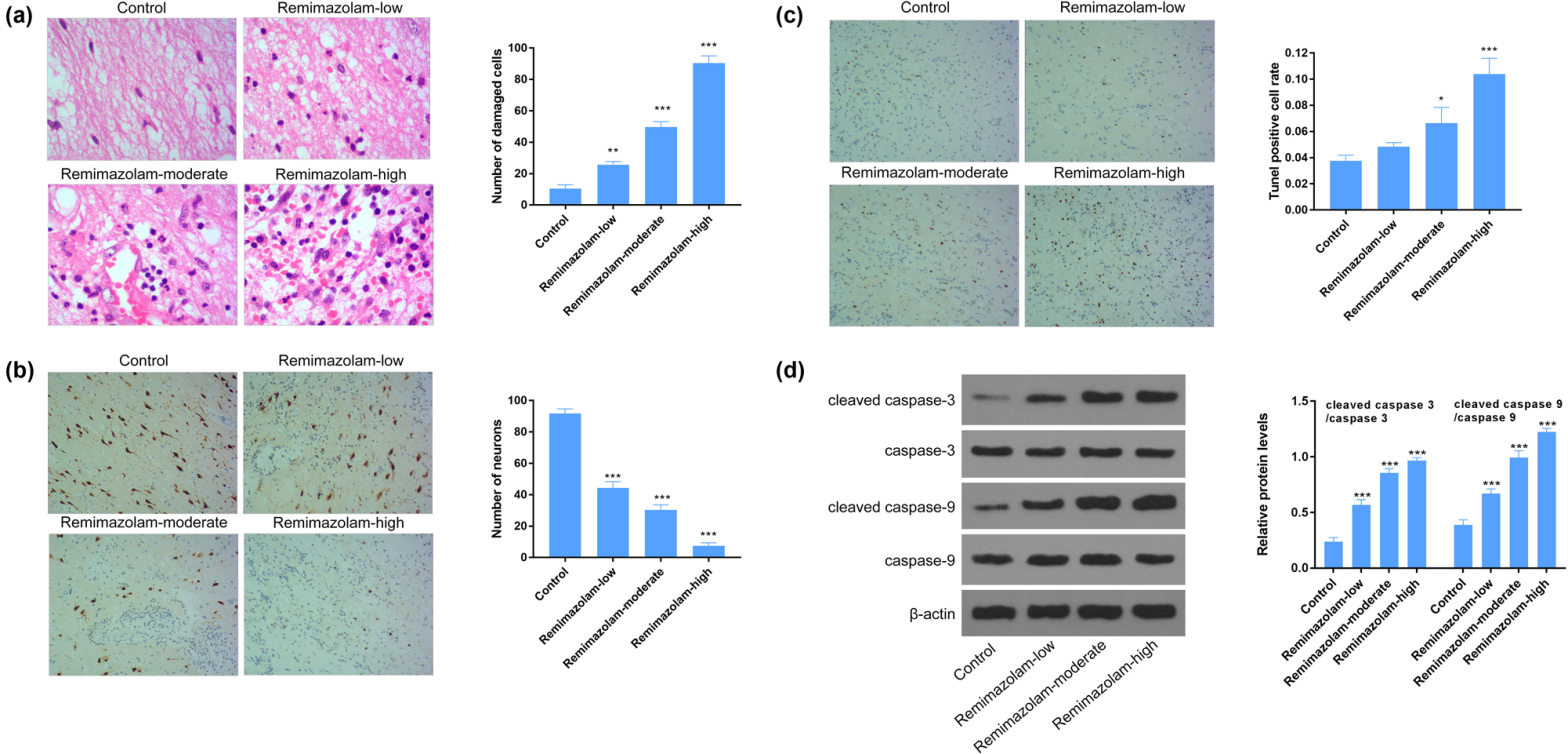
Remimazolam induced neuronal cell loss in mouse brain. (a) Representative images of neurons showing the damaged cells, right panel shows the quantifications of cells, isolated from mouse cortex. (b) Remimazolam mediated loss of neurons in brain cortex. Right panel shows the quantifications of neurons. (c and d) The apoptosis of neuronal cells in mouse brain, assessed by flow western blotting. Quantifications of protein levels. Experiments (*n* = 3). Data represent mean ± SEM. *P < 0.05, **P < 0.01, ***P < 0.001.

Next, we tested the effect of remimazolam on neuronal cells loss, and we observed that remimazolam induced significant neuronal loss in mouse cortex region of brain in a dose-dependent manner (Figure 1a). Then we studied remimazolam mediated morphological changes of neurons, that whether remimazolam induced morphological changes of neurons. H&E stain shows significant change of cortex neuronal morphology (Figure 1b). Next, to determine the role of remimazolam in cellular apoptosis, we performed terminal deoxynucleotidyl transferase-mediated deoxyuridine triphosphate nick end labeling assay and detected significant DNA fragments. We also analyzed a significant increased expression in C3 and C9. The significant higher expression of C3 and C9 suggest an apoptosis condition (Figure 1c and d). Taken together, these results imply that remimazolam induced pathological conditions by inducing neuronal loss and apoptosis.

### 4.2 Remimazolam regulated phosphorylation of tau protein

**Figure 2:**
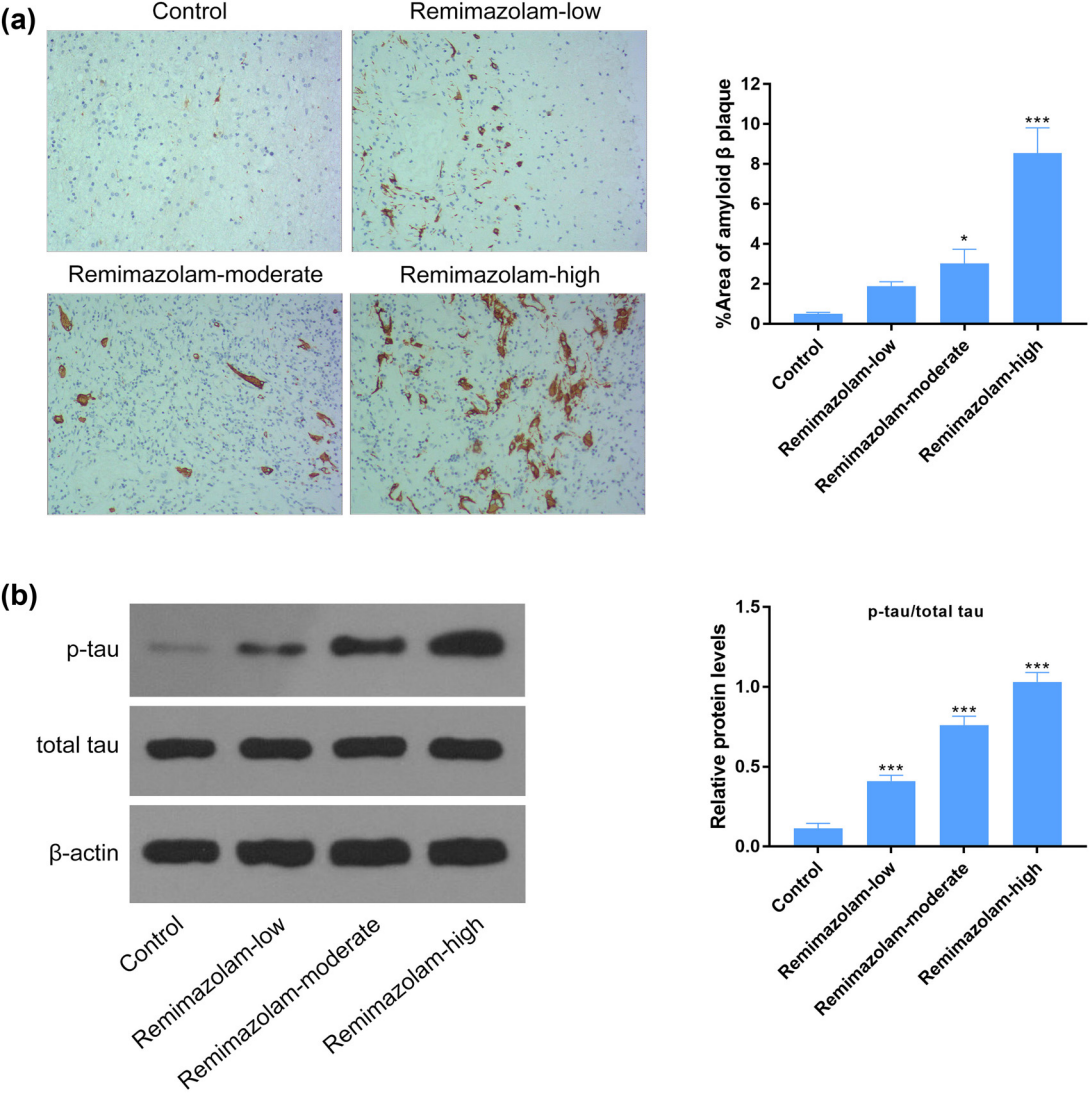
Remimazolam mediated hyper-phosphorylation of tau proteins in mouse brain. (a) Significant depositions of amyloid β plaque in remimazolam treated mice group assessed by immunohistochemistry. (b) Remimazolam significantly induced the activation of tau protein and phosphorylated tau in mouse brain, compared to control. Right panel shows the quantifications. Quantifications of protein levels. Experiments (*n* = 3). Data represent mean ± SEM. *P < 0.05, **P < 0.01, ***P < 0.001.

Anesthetic drugs have been reported to have its effect on phosphorylation of tau protein to induce anxiety or depression. We found remimazolam mediated increase in tau protein expression assessed by western blotting and confirmed that phosphorylated form of tau protein was hyper-phosphorylated in the presence of remimazolam compared to control, suggesting a vital role of repetitive doses of remimazolam in inducing depression as shown in Figure 2a. Next, we tested the effect of remimazolam on the deposition of amyloid β plaque. Amyloid β plaque is one of the common biomarkers of AD. None of the studies highlighted the role of remimazolam-mediated regulation of amyloid β plaque deposition. Therefore, we were also interested to dissect the role of remimazolam-associated regulation of amyloid β plaque. We found significantly higher level of amyloid β plaque deposition in mice treated with remimazolam, assessed by immunohistochemistry (Figure 2b).

### 4.3 Remimazolam induced glutamate excitotoxicity in mouse brain

**Figure 3:**
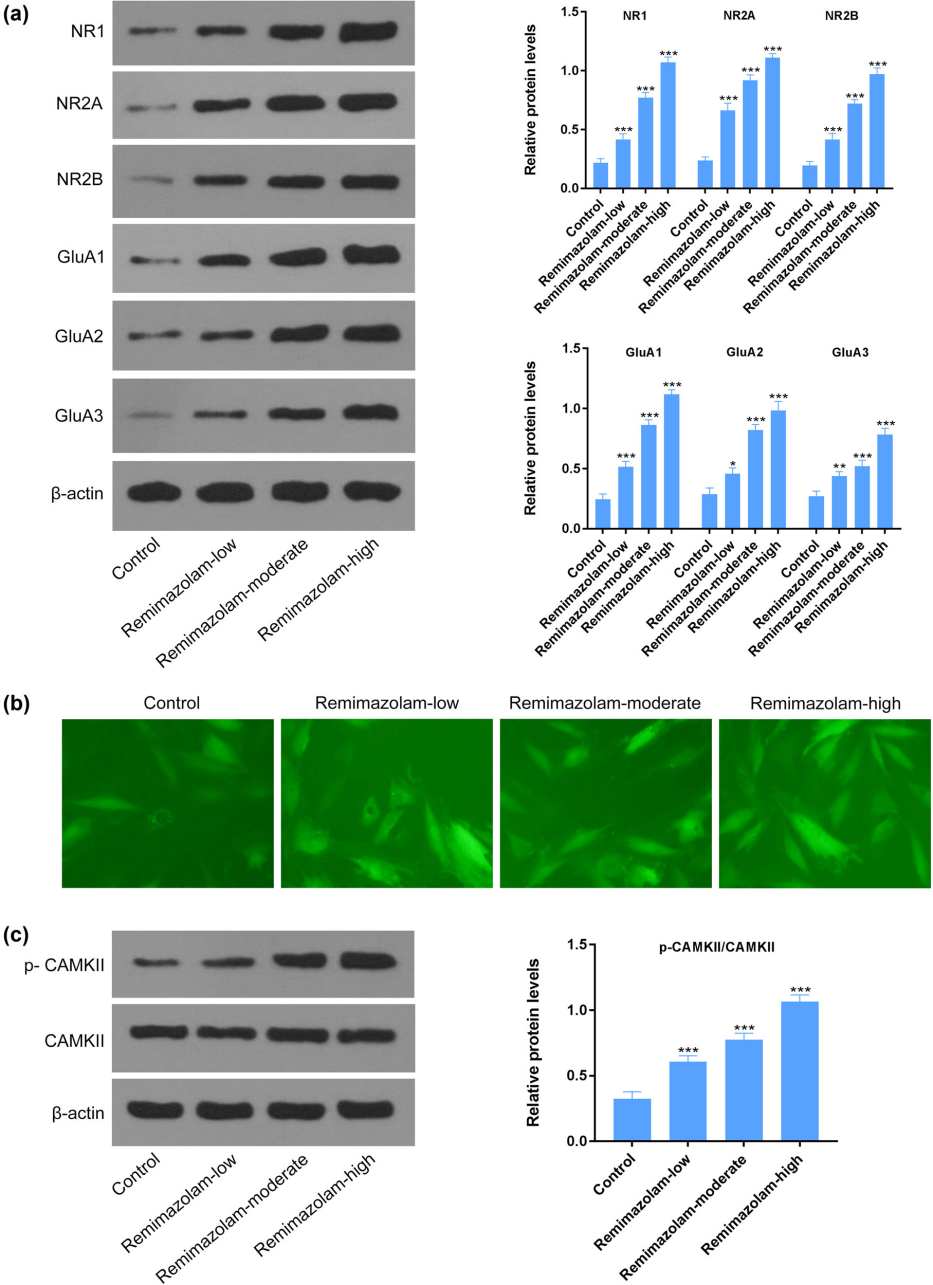
Remimazolam induced glutamate excitotoxicity by increasing Ca^2+^ influx. (a) Remimazolam increased expression of glutamate receptor NMDA or AMPA receptors. (b) Remimazolam mediated increased calcium influx in treated mice. (c) Remimazolam induced activation of CAMKII in treated groups. Quantifications of protein levels. Experiments (*n* = 3). Data represent mean ± SEM. *P < 0.05, **P < 0.01, ***P < 0.001.

Glutamate excitotoxicity is considered to be one of the most common factors for neuronal cell death due to the buildup of extracellular glutamate concentrations [21]. When the glutamate concentration increases, it further binds and activates glutamate receptors [32]. We found that remimazolam induced the activation of glutamate receptors NR1, NR2A, NR2B, GluA1, GluA2, and GluA3 assessed by western blotting, suggesting high extracellular glutamate concentrations (Figure 3a). Increased expression of glutamate receptors has been reported to be associated with Ca^2+^ influx [32]. Next, we wanted to see the expression of CaMKII, which gets activated in the presence of high cellular calcium level. We found a significant higher expression of total and phosphorylated CaMKII through western blotting (Figure 3b and c), suggesting an influx of calcium ions through glutamate receptors.

### 4.4 Remimazolam induced behavioral changes in mice

**Figure: 4:**
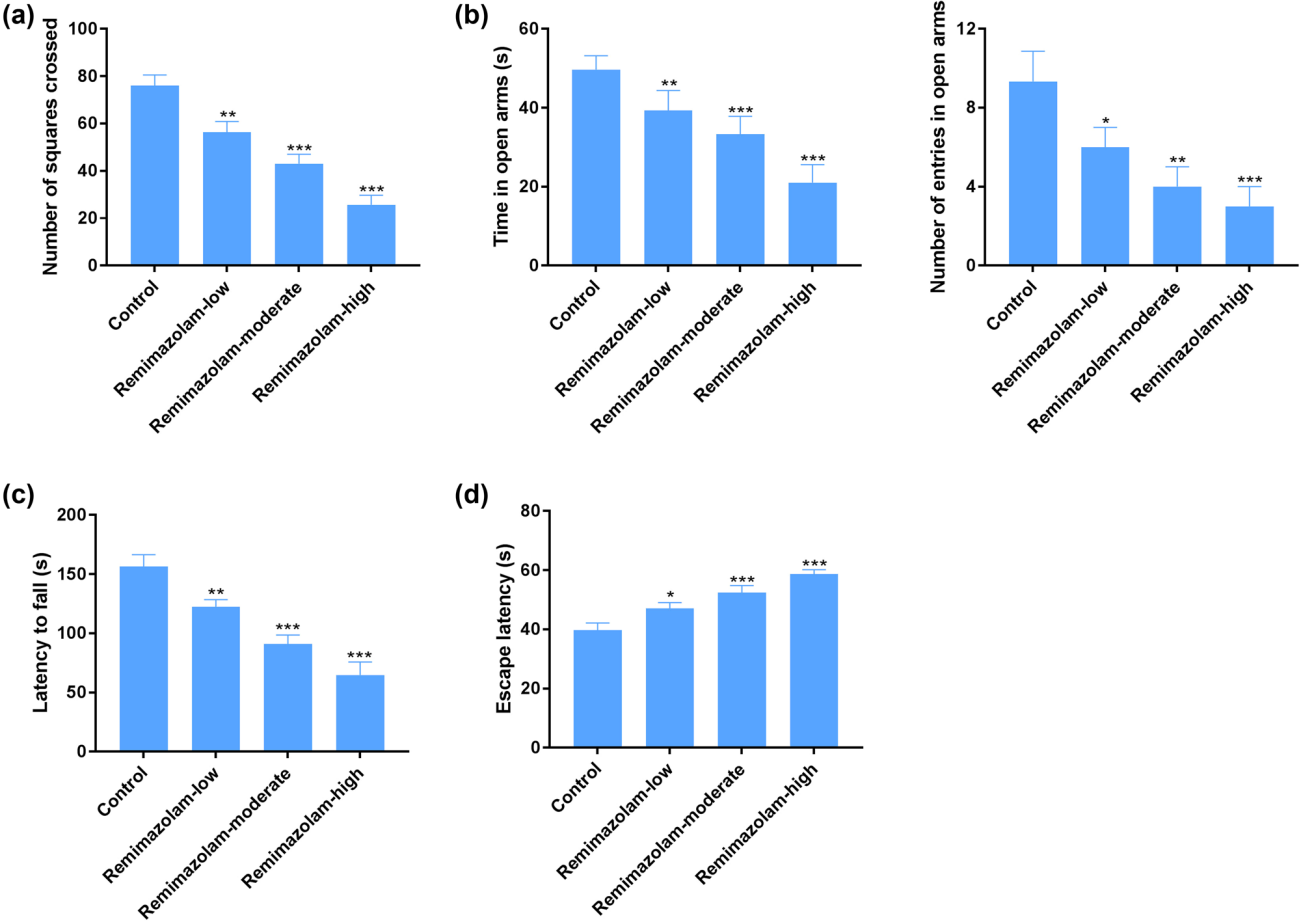
Remimazolam mediated changes in cognitive functions. (a) Open field test shows the significant difference of treated mice compared to control. (b) EPM test shows the anxiety-related behavior of rodent models in treated groups. (c) Rota rod test shows the impairment in motor learning skills of disease model group compared to control. (d) Morris water maze shows impaired cognitive functions in treated group compared to control. Quantifications of protein levels. Experiments (*n* = 8). Data represent mean ± SEM. *P < 0.05, **P < 0.01, ***P < 0.001.

Anesthetic drugs have been demonstrated to induce behavioral changes in mice or rats [33,34]. Remimazolam is reported to induce sedative effects [22]. Here, we tested whether or not remimazolam is associated with anxiety- and memory-related behavioral tests. We found that repetitive dosages (10, 15, and 20 mg/kg) of remimazolam induced anxiety behavior, assessed by open field and elevated plus maze test (Figure 4a and b). Mice treated with remimazolam showed significant difference compared to control group, as the treated group showed less motivation to explore the open field arena compared to control group. We also assessed rota rod and Morris water maze test, which are crucial for identifying learning and motor skills. Remimazolam-treated mice group showed a significant difference on rota rod and Morris water maze test compared to the control group (Figure 4c and d).

### 4.5 Remimazolam mediated regulation of glial cells in mice brain

**Figure 5:**
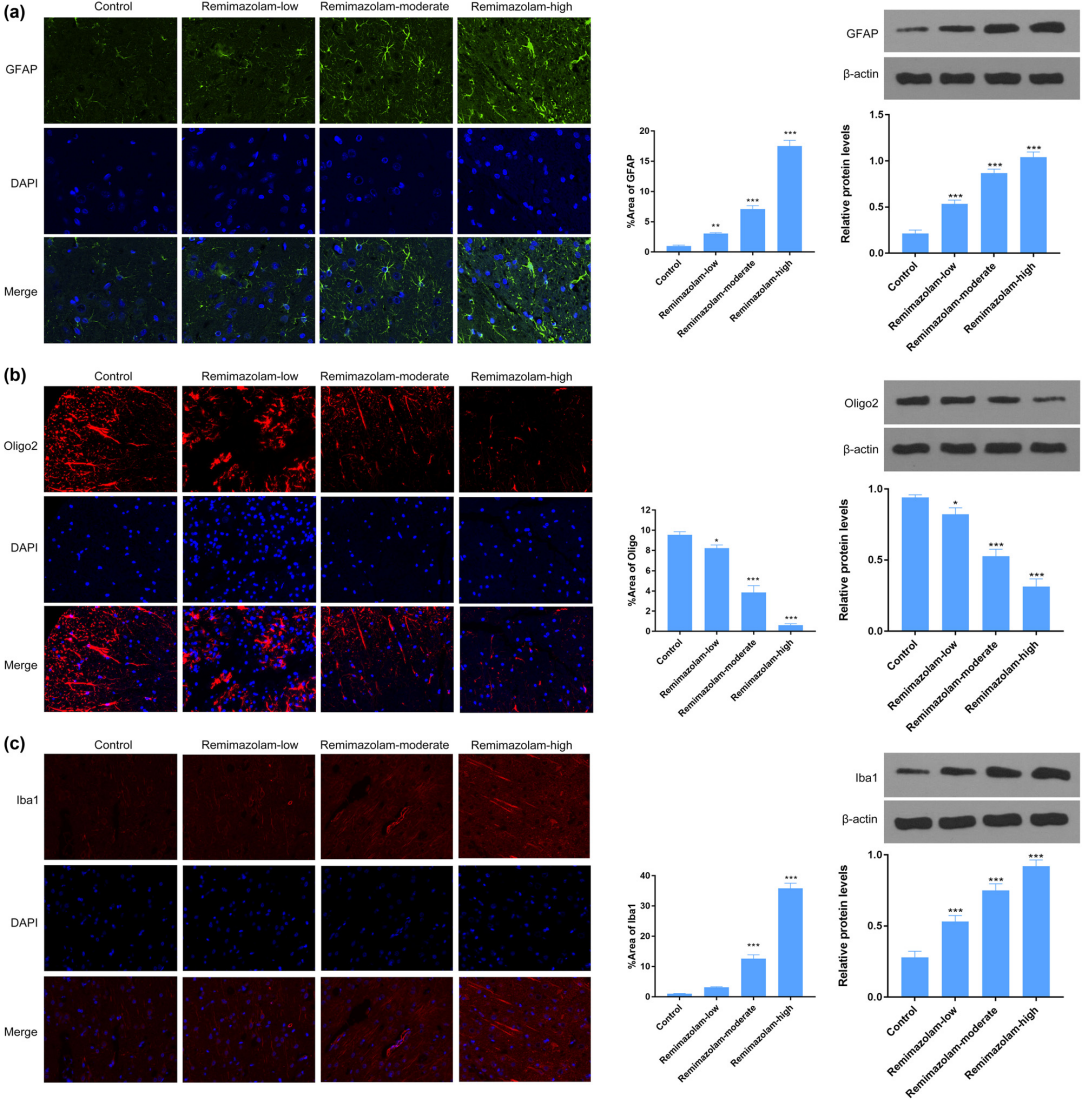
Remimazolam induced changes in CNS cells. (a) Remimazolam mediated activation of astrocytes in brain cortex, treated group showed significant astrocytes activation. (b) Remimazolam significantly increased the number of oligodendrocyte cells in brain cortex. (c) Activation of microglial cells in brain cortex in treated group compared to control. Right panels show quantifications of protein levels. Experiments (*n* = 3). Data represent mean ± SEM. *P < 0.05, **P < 0.01, ***P < 0.001.

Glial cells are crucial in maintaining the homeostasis of brain environment. Remimazolam effects on glial cells have not been reported before. Astrocyte activation is commonly reported in many neurodegenerative disorders and is considered a reliable marker. Therefore, we wanted to see the effect of remimazolam on astrocyte. Here, we report that repetitive doses of remimazolam induced the activation of astrocytes, thus reflecting a pathological condition. We used different concentrations of drug (10, 15, and 20 mg/kg). Highest concentration of remimazolam-treated mice showed significant activation of astrocytes compared to control (Figure 5a). Oligodendrocytes are the myelinating cells and play a vital role in the formation of myelination sheath around axons. Next, we asked whether or not remimazolam regulated oligodendrocyte. We found that 20 mg/kg concentration of remimazolam induced significant reduction in oligodendrocyte number assessed by Oligo^2+^ marker in the mouse cortex (Figure 5b). We also found significant microglial activation which plays an important role in the CNS, when treated with 20 mg/kg concentration of drug (Figure 5c). Taken together these results suggest that remimazolam accelerated pathological conditions in the mouse brain, by activating astrocytes and microglial cells.

## 5 Discussion

Present study was designed to investigate the effect of remimazolam on glial cells, memory function, anxiety, motor behavior, and its possible molecular mechanism including tau-phosphorylation, Aβ-plaque, and neuronal loss in inducing memory deficits. This study showed that remimazolam induced cognitive impairment which was associated with hyper-phosphorylation of tau protein followed by aggregation of tau protein and neuronal loss in cortex. We also showed remimazolam mediated regulation of glial cells. Glutamate excitotoxicity was also increased in remimazolam-treated animals, which had potential role in neuronal apoptosis.

Several lines of evidence demonstrated the role of anesthetic drugs in impairment of memory and cognitive functions [11,14–16,24]. It is reported that administration of anesthetic drug during development phase of nervous system produced long-term neuronal and behavioral changes, and impairments in memory functions [35]. Evidence showed that ketamine as anesthetic as well as sub-anesthetic doses impaired acquisition and consolidation, spatial and recognition memory. Dexmedetomidine had also showed consistent effects of memory deficit in rodents [36].

Motor activity was decreased post-24 h of ketamine administration [37] but did not alter after 48 and 72 h of drug administration [38]. In our study remimazolam produced impairment in motor functions. Anesthetic drugs such as ketamine and midazolam are used alone and in combination as premedication before surgery to reduced anxiety and stress in clinical studies [39,40]. Preclinical studies showed both anxiogenic and anxiolytic effects of anesthetic drug ketamine at sub-anesthetic doses [41–44]. Ketamine administered 24 h prior to light and dark test produced anxiogenic effects [37]. Here, in this study we tested whether or not remimazolam is associated with anxiety- and memory-related behavioral tests. We found that repetitive dosages (10, 15, and 20 mg/kg) of remimazolam induced anxiety behavior, assessed by open field and elevated plus maze test. Remimazolam-treated mice showed significant difference compared to the control group, as the treated group showed less motivation to explore the open field arena compared to the control group. Rota rod and Morris water maze test also assessed the ability of learning and motor skills. We found that mice treated with remimazolam showed a significant difference on rota rod and Morris water maze test compared to the control group. Repeated administration of remimazolam produced anxiolytic effects in elevated plus maze test in mice. In line with previous studies on anesthetic drugs our study showed that repeated remimazolam produced memory impairing effects.

Astrogliosis is a common biomarker for neurodegenerative disorders and has been reported in several studies [4,5,45]. In this study, we found that astrocytes were significantly enhanced in remimazolam-treated group compared to control, which suggests a pathological condition caused by repetitive doses of remimazolam. Brambrink et al. showed that isoflurane induced apoptosis of oligodendrocyte cells in post-natal 6 days [46]. However, the effect of remimazolam on oligodendrocyte cells is not reported yet. Here, we show remimazolam mediated death of oligodendrocyte cells. Remimazolam significantly reduced the number of oligodendrocytes, which is in line with previous studies of anesthetic drugs. Microglial cells play a crucial role in eliminating damaged neuronal cells or synapses and maintain the homeostasis of CNS [47], while its activation has been reported in pathological conditions [6]. We report that repetitive doses of remimazolam induced significant microglial activation. Previous studies demonstrated that administration of anesthetic drugs induced abnormal tau protein phosphorylation and consequent aggregation/formation of neurofibrillary tangles [26,48]. Formation of neurofibrillary tangles in brain associated with memory deficit and AD [49,50]. Studies suggest that anesthesia mediated tau hyper-phosphorylation induced memory impairment [51].

Neuronal loss in cortex and abnormal tau protein phosphorylation were also observed in animals treated with remimazolam. These results suggest that cognitive deficit were associated with neuronal loss and accumulation of protein in brain due to hyper-phosphorylation of tau protein. Apoptosis of neurons is recognized as harmful effect of anesthetic drugs [12,52–54]. Accumulated data showed that prolonged or high doses of propofol administration *in vitro* and *in vivo* exhibited enhanced neuronal apoptosis [55,56]. Neurons but not astrocytes or oligodendrocytes were more prone to apoptosis when exposed to propofol [57]. Previous studies reported that propofol and isoflurane exposure to rhesus macaques exhibited neuronal and oligodendrocyte apoptosis [58,59]. Consistent with previous research studies current study also showed that remimazolam repetitive treatment decreased oligodendrocyte cells in brain cortex.

Glutamate excitotoxicity is one of the common factors of neuronal death due to the buildup of extracellular glutamate concentrations, which further facilitates the influx of excessive ions and leads to cell death [21]. It refers to the pathological condition in which several changes in cellular events take place. Evidence showed that anesthetic drug such as ketamine and isoflurane produced neuronal cell death via mitochondrial pathway by increasing activity of different caspase proteins and reactive oxygen species production [56,60]. Studies also showed cell apoptosis due to elevated cytosolic calcium [61]. In the current study, remimazolam induced apoptosis through glutamate excitotoxicity by increasing Ca^2+^ influx.

Overall these results showed that remimazolam produced memory dysfunction by producing neuronal apoptosis via glutamate excitotoxicity and exhibited amyloid β plaque in brain via abnormal phosphorylation of tau protein. Remimazolam had anxiolytic effects but it produced impairment in motor function. This study may be helpful to improve the therapeutic effects of anesthetic drugs.
